# Influence of Silver Nanoparticles on the Biological Indicators of Haplic Chernozem

**DOI:** 10.3390/plants10051022

**Published:** 2021-05-20

**Authors:** Sergey Kolesnikov, Natalia Tsepina, Tatiana Minnikova, Kamil Kazeev, Saglara Mandzhieva, Svetlana Sushkova, Tatiana Minkina, Mahmoud Mazarji, Rupesh Kumar Singh, Vishnu D. Rajput

**Affiliations:** 1 Academy of Biology and Biotechnology Named D.I. Ivanovsky, Southern Federal University, 344090 Rostov-on-Don, Russia; kolesnikov1970@list.ru (S.K.); cepinanatalia@yandex.ru (N.T.); loko261008@yandex.ru (T.M.); kamil_kazeev@mail.ru (K.K.); msaglara@mail.ru (S.M.); terra_rossa@mail.ru (S.S.); tminkina@mail.ru (T.M.); mahmoudmazarji@gmail.com (M.M.); 2 Centro de Química de Vila Real, Universidade de Trás-os-Montes e Alto Douro, Quinta de Prados, 5000-801 Vila Real, Portugal; rupeshbio702@gmail.com

**Keywords:** pollution, haplic chernozem, number of soil bacteria, enzymatic activity, phytotoxicity, integrated index of biological state, biotesting, ecotoxicity

## Abstract

In recent years, silver nanoparticles (AgNPs) are increasingly used in various industries due to their antibacterial properties, which lead to an increase in pollution of the environment and soil ecosystems. However, the ecological effects of soil pollution by AgNPs were poorly studied than that with AgNPs of other metal-based NPs. The aim of this study is to assess the influence of AgNPs on the biological properties of Haplic Chernozem. Silver was introduced into the soil in the form of AgNPs with a concentration of 0.5; 1; 5; 10; 50, and 100 mg/kg in laboratory conditions. The influence of AgNPs on the biological properties of Haplic Chernozem was assessed 30 days after contamination. The degree of reduction in biological properties depends on the AgNPs concentration in the soil. This study showed that the sensitivity to contamination by AgNPs in the total number of bacteria and enzymatic activity was more than that in the abundance of bacteria of the genus *Azotobacter*. The integrated index of biological state (IIBS) of Haplic Chernozem was decreased by contamination with AgNPs. Silver nanoparticles in the concentration of 10 mg/kg caused a decrease in the indicator by 13% relative to the control. It also decreased IIBS by doses of 50 and 100 mg/kg by 22 and 27% relative to the control. All used biological indicators could be used for biomonitoring, biodiagnosis, bioindication, and regulation of ecological condition of soil contamination by AgNPs.

## 1. Introduction

Silver is a precious metal that has a multidirectional effect on living organisms. The degree of exposure depends on the concentration and forms of the metal. Silver protects plants from fungal, viral, and microbial diseases [1]. The bactericidal, fungicidal, virucidal effect of silver in plants is explained by the fact that its ions penetrate into the cell-causative agent of the disease and block its enzymes [2,3]. In addition, silver can interact with thymine and guanine in the deoxyribonucleic acid (DNA) molecule of bacteria, fungi, and viruses, which disrupts the DNA functions, inhibits growth and reproduction [4]. The form of the chemical compound of silver significantly affects the ecotoxicity.

Nanomaterials of silver in the form of silver nanoparticles (AgNPs) are more toxic than in the form of bulk [5]. Silver nanoparticles may be released into the environment by several routes, including during their synthesis, incorporation into other goods, translocation and recycling or disposal of these goods, and are of particular interest among other nanomaterials. Due to antibacterial properties, AgNPs are widely used in food, textile, construction, medical, cosmetic, pharmaceutical, and other industries [6,7,8,9]. According to the forecasts, the production of AgNPs is expected to reach approximately 800 tons by the year 2025 [10], which will create huge risks of environmental and soil pollution [11,12,13]. Silver nanoparticles are novel and promising functional materials due to their antibacterial, antifungal, antiviral, and anti-inflammatory activity [5].

It was found that the AgNPs were affected plants [14,15,16,17], soil microorganisms [18,19,20], and the activity of soil enzymes [21,22,23,24]. In addition, silver is known to accumulate in plants, mainly in root tissues [25]. In some cases, the introduction of AgNPs promotes the growth of the root length of various crops, including tomato (*Lycopersicones culentum* Mill), radish (*Raphanus sativus* L.), wheat (*Triticum aestivum* L.), beans (*Phaseolus vulgaris* L.), corn (*Zea mays* L.), watermelon (*Citrullus lanatus* (Thunb.) Matsum. and Nakai), and zucchini (*Cucurbita pepo* L.) [26,27,28,29,30,31]. However, the ecological effects of soil pollution with AgNPs were studied poorly compared to the nanosized of other metal-based NPs, such as iron, nickel, zinc, and copper [32,33,34]. Therefore, it seems relevant to identify the influence of AgNPs on the biological properties of the Haplic Chernozem.

The assessment of the ecotoxicity of AgNPs on soil microorganisms, the activity of enzymes, and phytotoxicity indicators are allowed to understand the influence of Ag on the soil ecosystems. Oxidoreductases (catalase, dehydrogenase, peroxidases, and polyphenoloxidases) are functionally necessary for the decomposition of pollutants, the transformation of organic matter, and the maintenance of the metabolism of microorganisms [35,36]. Catalase activity decrease with soil contamination with petroleum hydrocarbons and heavy metals [37,38,39]. Catalase activity is related to the metabolic activity of aerobic organisms and is often used as one of the indicators of soil fertility. The phytotoxic properties of the soil were analyzed in terms of the intensity of initial seed growth of rate (germination rate), and length of the roots of radish seedlings (*Raphanus sativus* L.). 7 The use of these biological indicators will open up many opportunities for both cleaning and restoring soil fertility after contamination. The purpose of this investigation was to establish the influence of AgNPs on the biological indicators (enzymatic activity, microbiological and phytotoxicity indicators) of Haplic Chernozem.

## 2. Materials and Methods

### 2.1. Study Site

The soil for the study was classified as Haplic Chernozem [40]. The study site was located within the Botanical Garden of the Southern Federal University, Rostov on Don, Russia (47°14′17.54″ N, 39°38′33.22″ E). The soil was sampled from fallow land covered by natural herbaceous vegetation and perennial grasses [41]. This soil type was characterized as a heavy loam granulometric composition, an average organic matter content of 3.7% and pH_H2O_ = 7.8 (nearly neutral). For this experiment, Haplic Chernozem was sampled (soil layer of 0–20 sm.) since heavy metals were deposited at this site [42].

### 2.2. Experimental Details

The objective of this study was to provide a comprehensive assessment of the influence of AgNPs on the enzymatic activity of the soil, microbial, and phytotoxicity indicators based on Ag concentration. For the modeling experiment, AgNPs (Sigma-Aldrich Ltd.,St. Louis, Missouri, USA) were used with a size of 10 nm (0.02 mg/mL), supplied in 2 mM sodium citrate as a stabilizer. The conditionally permissible concentration (CPC) values were taken equal to 3 background silver concentrations in the soil as CPC of most heavy metals and metalloids [43].

The silver content in contaminated soils often reached 35 to 7000 mg/kg (the soils of ore) [44,45]. The background silver concentrations in Haplic Chernozem were 0.303 mg/kg. The silver content in soils was defined by the Inductively Coupled Plasma Mass Spectrometry (ICPMS) ELAN-DRC-e instrument (Perkin Elmer). The measuring range of ICPMS was from 10^−6^ to 0.1% in the laboratory of A.P. Karpinsky Russian Geological Research Institute, St. Petersburg (VSEGEI/CGMW). Accordingly, the CPC was taken equal to 1 mg/kg. Silver was introduced into the soil in the amount of 1.5, 3, 15, 30, 150, and 300 background concentrations (0.5, 1, 5, 10, 50, and 100 mg/kg, respectively). Silver was added in the form of solutions (in distilled water) AgNPs of specified concentrations. Soil samples weighing 1 kg were added with solutions of AgNPs of concentrations: 0.5, 1, 5, 10, 50, and 100 mg/kg. The soil was placed in vegetative pots. Haplic Chernozem with AgNPs was incubated under temperature (22–25 °C) and soil moisture (60%). The influence of AgNPs on the biological properties of Haplic Chernozem in the model experiment was assessed 30 days after contamination.

### 2.3. Measurement Procedures

Laboratory studies of biological indicators were performed using methods (Table 1). It was efficient to use sensitive biological indicators to diagnose soil conditions after chemical pollution [43,46,47]. The used set of indicators gave an informative estimate of the biological processes taking place in the soil and the ecological state of the ground. The total number of bacteria, the abundance *Azotobacter* sp., the activity of catalase and dehydrogenases, and the phytotoxic properties of the soils (germination rate and length of roots of garden radish (*Raphanus sativus* L.)) were investigated.

#### 2.3.1. Measurement of Soil Organic Matter and pH

In Haplic Chernozem, before contamination, AgNPs were determined soil organic matter content (%) and pH. The potassium dichromate method (NY 1121.6 2006) was employed to determine organic matter content in soil samples [48]. Soil pH was measured using an electrode potentiometer in distillate water, in the ratio of 1 part soil to 2.5 parts of water (*w*/*v*).

#### 2.3.2. Measurement of the Total Number of Bacteria

The total number of bacteria in the soil reflects the state of reducers in the ecosystem [32]. The total number of bacteria in the soil was determined by the luminescence microscopy method considering the number of soil bacteria after staining with acridine orange dye [49]. Acridine orange is a fluorochromatic dye that binds to nucleic acids bacteria, and other cells. Under the influence of ultraviolet radiation, acridine orange stains ribonucleic acid (RNA) and single-stranded DNA in orange color (as soil particles), double-stranded DNA in green (as bacterial cells). After incubation, the fresh soil was dried, and a soil suspension (soil:water, 1:100) was prepared. On prepared glasses (defatted and sterilized), 10 μL of soil suspension was placed, air-dried (air temperature—22–24 °C), and dried in a burner flame (duration 3–5 s). After that, the glasses were stained with a solution of acridine orange dye (dilution of the solution of acridine orange dye, 1:10,0000) for 20 min. The glasses were washed to remove excess dye and dried in the air. The glasses were viewed under a Carl Zeiss Axio Lab A1 microscope at a magnification of X40 (20 bacterial cells of counting fields).

#### 2.3.3. Measurement of Bacteria of the Genus *Azotobacter*

Bacteria of *Azotobacter* sp. abundance was traditionally used to indicate chemical pollution of the soils [50]. The abundance of bacteria of the genus *Azotobacter* sp. was determined by the method of fouling lumps on Ashby medium. To assess the number of bacteria, Ashby’s medium was prepared. The medium was poured into Petri dishes and lumps of moistened soil (25 pieces per 1 dish, temperature of incubation, 22–25 °C) were stirred. These operations were performed in an abacterial air-box BAVnp-01—“Laminar-S”. The number of fouling lumps was counted 14 days after the start of the experiment. Counting of soil lumps overgrown with *Azotobacter* sp. mucus was carried out relative to the control.

#### 2.3.4. Measurement of the Activity of Catalase and Dehydrogenases

The activity of catalase and dehydrogenases estimated the potential biological activity in soils. Oxidoreductases (catalase and dehydrogenases) were more sensitive to chemical pollution than other enzymes [51,52]. Catalase activity was determined according to Galstyan’s [53]. The enzyme activity was determined by the gasometric method by the rate of decomposition of 5% hydrogen peroxide after contact with the soil (temperature, 20–22 °C). Dehydrogenases were determined according to Galstyan’s method modified by Khaziev [53]. The activity of dehydrogenases was determined by the conversion of TPC to TPF. The optical density of the colored solutions was determined spectrophotometrically on a PE 5800VI spectrophotometer at a wavelength of 540 nm.

#### 2.3.5. Measurement of Germination Rate and Length of Roots of Radish

Soil phytotoxicity was investigated by the germination rate of radish (*Raphanus sativus* L.) and length of roots in growth chamber Binder KBW 240 [54]. To assess soil toxicity using plants, garden radish (*Raphanus sativus* L.) was used. Compared to other plant test objects, radish had a fast response to soil nutrients and moisture [55]. Germination rate and root length of radish were the most informative of the many indicators of soil phytotoxicity [37,56,57,58].

After incubation of the soil with AgNPs for 30 days, the soil was placed in a Petri dish. Radish seeds were planted in a Petri dish of the soil mass—25 seeds in Petri dishes in conditions of moisture 60% and temperature of 24–25 °C. After 7 days of the experiment, the radish was pulled out of the soil and the germination of seeds and the length of the radish roots were determined. Germination rate was assessed by the number of germinated radish seeds in 7 days of the experiment (after the appearance of 2 or more leaves).

### 2.4. Data Analysis

The results of soil bacteria were expressed in 10^9^ bacteria in gram of soil dry weight (Equation (1)):

(1) $$M=\frac{b\times A\times H\times T}{P}$$

where M—number of cells per 1 g of fresh soil; A—the average number of cells within one field of vision; b—coefficient magnification factor (b = 4); H—dilution index; T—conversion factor in billions of bacteria per 1 g of soil (T = 10^10^); P—the area of the field of vision, µm^2^.

The indices of the intensity of the initial growth of radish seeds (length of radish roots) were calculated as the average triplicate [51]. The indicator of seed germination rate was calculated using Equation (2):

(2) $$G=\frac{n_{1}+\ldots +{\;n}_{m\;}}{m}$$

where G—seed germination rate; n_1_—number of the seed of 1st replicate; n_m_—number of the seed of m replicate; m—quantity of replicates.

Integrated index of biological state (IIBS) of the soil was allowed to give an integral assessment of the condition of soils after any chemical pollution [37,51]. For the calculation of IIBS, the value of each of the above indicators on the control (in unpolluted soil) was taken as 100%. The percentages in other experimental variants (in polluted soil) were expressed as a percentage relative to control. For the IIBS condition maximum value of each index (100%) was chosen from array data, and in reference to the value of this index was expressed for other variants of experiments by Equation (3):

(3) $$B_{1\;}=\frac{B_{x}}{B_{\max}}\times 100\%$$

where B_1_—is the relative score of the indicator; B_x_—the actual value of the indicator; B_max_—is the maximum value of the indicator.

The relative values of several mostly informative indices of soil biological condition such as the activity of catalase and dehydrogenases, total number of bacteria, abundance of *Azotobacter* bacteria, length of roots, germination of radish seeds were summed.

Thereafter, the average assessment point of studied indices was calculated for each variant by Equation (4):

(4) $$B=\frac{B_{1}+B_{2}+\ldots +B_{n}}{N}$$

where B—average estimated score of indicators; B_1_…B_n_—the relative score of the indicator; N—is the number of indicators.

The integral index of the soil biological condition (IIBS) is calculated by Equation (5):

(5) $$\mathrm{IIBS}=\frac{B}{B_{\max}}\times 100\%$$

where B—is the average estimated score of all indicators; B_max_—is the maximum estimated score of all indicators.

During diagnostics of the contamination value of each index in non-contaminated soil, it was taken as 100%. With reference to its value of the same index in the contaminated soil was expressed in percent, then the average value of 6 selected biological indicators for each experiment was determined. The obtained value IIBS was expressed as a percentage concerning the control (to 100%). The methodology used allowed integrating the relative values of different indicators, which cannot be integrated since they have different units of measurement.

The degree of sensitivity of biological indicators was assessed by the degree of decrease in the values of the biological indicator compared to the control. The more the value of the biological indicator decreased from the control (100%), the more sensitive this biological indicator.

The informative value (informativeness) was assessed by the tightness of the correlation between the biological indicator and the concentration of silver in the soil. The close the correlation coefficient is R = −1, the higher the information content of this biological indicator.

### 2.5. Statistical Analyses

An analysis of the rate of variation (standard deviation) at *p* ≤ 0.05 to determine the reliability of the results. Data were means of triplicate. Statistical data processing was carried out using Statistica 12.0 and Python 3.6.5 Matpolib package. The correlation nonparametric Spearman’s coefficient was calculated between the concentration of AgNPs and the average of biological indicators.

## 3. Results

### 3.1. Influence of AgNPs on Microbiological Conditions of Soil

The influence of AgNPs on the total number of bacteria of Haplic Chernozem is presented in Table 2. The statistically reliable decrease of the total number of bacteria was not observed when AgNPs were introduced into the soil in the amount of 0.5 mg/kg (*p* < 0.05). Doses of 1 and 5 mg/kg caused a decrease in the total number of bacteria by 11 and 22% (*p* < 0.05), respectively, relative to the control values. The inhibition of the total number of bacteria was observed by 27% relative to the control when AgNPs were introduced into the soil in the amount of 10 mg/kg (*p* < 0.05). The indicator decreased by 51% relative to the control values at 100 mg/kg (*p* < 0.05).

The effects of AgNPs on the *Azotobacter* sp. abundance are shown in Table 2. Results indicate the statistically reliable decrease of the abundance of bacteria of the genus *Azotobacter* was not noticeable in the soil contaminated by AgNPs in the dose of 100 mg/kg (*p* < 0.05). The abundance of bacteria of the genus *Azotobacter* sp. in this study was the least sensitive of the studied biological indicators. At the same time, previous studies on contamination of Haplic Chernozem by silver nitrate showed a decrease in abundance by 23–24% relative to the control [32]. Haplic Chernozem was more resistant to AgNPs contamination in terms of the number of bacteria of the genus *Azotobacter* compared to the total number of bacteria. The total number of bacteria was a more informative and sensitive indicator to AgNPs contamination than the *Azotobacter* sp. abundance.

### 3.2. Influence of AgNPs on the Activity of Enzymes of Soil

Silver nanoparticles in the amount of 0.5, 1, 5, 10, and 50 mg/kg caused the decrease of the catalase activity by 18, 20, 24, 28, and 31% (*p* < 0.05) relative to the control (Table 2). The indicator decreased by 40% relative to the control values at 100 mg/kg. The activity of dehydrogenases of Haplic Chernozem were affected in all the applied AgNPs concentrations (Table 2), and it decreased 38, 41, 52, 59, 65, and 76%. at concentrations of 0.5; 1; 5; 10; 50 and 100 mg/kg applied AgNPs, respectively relative to the control values. The results show that enzymatic activity (activity of catalase and dehydrohenases) were the most affected by different concentration of AgNPs. At the minimum concentration of AgNPs (0.5 mg/kg), a inhibition in enzymatic activity by 18–38% relative to the control was observed.

### 3.3. Influence of AgNPs on Germination Rate and Root Length of Radish

The applied concentrations of AgNPs influence the germination rate and root growth of radish in various ways are shown in Table 2. A statistically unreliable stimulating effect of AgNPs in the amount of 0.5 and 1 mg/kg on the radish germination was noted. Whereas the statistically significant stimulating effect of AgNPs in the amount of 1 mg/kg on the length of radish roots was observed 21% relative to the control values. However, a dose of 50 mg/kg of AgNPs reduced the germination rate of radish by 29%. At the doses of 5, 10, and 50 mg/kg of AgNPs, the statistically reliable decreases were observed in the length of radish roots by 26–49%, relative to control values. The indicator for germination rate was decreased by 40% (*p* < 0.05) by AgNPs at a dose of 100 mg/kg. The radish root length decreased by 65% (*p* < 0.05) at the concentration of 100 mg/kg, relative to the control values. Even at a dose of 1 mg/kg, the length of radish roots decreased by 21%, which was an indicator of the sensitivity of this parameter. As the concentration of AgNPs increased, the degree of exposure increased.

### 3.4. Integrated Index of the Biological State of Soil Contaminated by AgNPs

**Figure 1:**
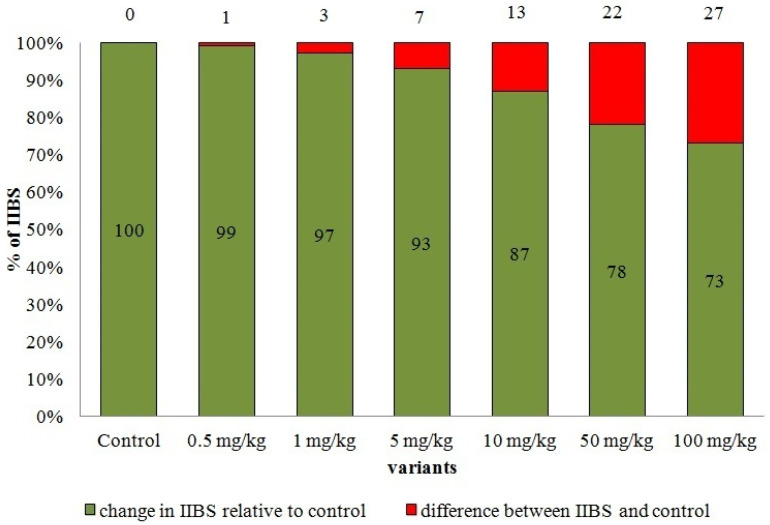
Change in IIBS of Haplic Chernozem by AgNPs pollution, % of control.

Silver nanoparticles in the concentration of 10 mg/kg caused a decrease in the indicator by 13% relative to the control (Figure 1). Silver nanoparticles decreased IIBS in concentrations 50 and 100 mg/kg by 22 and 27% (*p* < 0.05) relative to the control, respectively.

With an increase in the concentration of AgNPs (up to 100 mg/kg), IIBS decreased by 22–27%. The decrease in IIBS by more than 10% indicated serious disturbances in the functioning of the soil. IIBS was used as an indicator of disturbance in the ecosystem functions of the soil. With a decrease in IIBS by less than 5%, a disturbance in the ecosystem functions of the soil did not occur, but a decrease in the value of IIBS by 5–10% revealed a violation of information functions; by 10–25% biochemical, physicochemical, chemical, and integral functions; and more than 25% physical functions [43]. Then the higher the concentration of AgNPs, the higher the decrease of integral indicators of biological state (IIBS).

## 4. Discussion

The results of the previous studies showed that more small AgNPs (<10 nm) could permeate the cell directly and cause more damage, such as the intervention in DNA and protein synthesis, redox, and organoid functions [3,59]. These mechanisms are likely to lead to a decrease in dehydrogenases activity in the soil. The level of ecotoxicity of AgNPs depends not only on the dose of the Ag but also on the class of enzyme. In the current research, it was found that the activity of dehydrogenases is more sensitive to Ag pollution than that of activity catalase. The high sensitivity of activity of dehydrogenases to Ag was also confirmed by Yan et al. [24]. Previous research has found impacts of AgNPs on enzyme activities, microbial biomass, microbial diversity, nitrification, and decomposition of organic matter of soil [19,20,22,60].

There are studies devoted to the ecotoxicological effect of AgNPs on the activity of soil of acid and alkaline phosphatase, β-glucosaminidase, β-glucosidase, arylsulfatase, urease, dehydrogenases, phenoloxidases, and arylamidase [23,24,25,61]. Arylamidase and phenoloxidase activity was inhibited by high doses of 100 mg/kg of AgNPs [62]. Silver nanoparticles inhibited the activity of soil exoenzymes—dehydrogenases, urease, acid phosphatase, and alkaline phosphatase in the rhizosphere of such wetland plants as *Iris wilsonii* L., *Typha orientalis* L. and *Arundo donax* L. [22]. It was found that the activity of dehydrogenases is more susceptible to AgNPs than the activity of catalase. At AgNPs concentration of 100 mg/kg, the dehydrogenases activity decreased by 92% after contamination [25].

Earlier, a decrease in rice seed germination under the influence of AgNPs was noted [63]. Stimulation of the root length of radish, wheat, beans, and corn was also identified by other authors [29,31]. Silver nanoparticles had a toxic effect on the germination, growth, and development of rice seeds [64]. Silver nanoparticles accumulated in the roots of marsh plants (*Phragmites australis* L.) and also exerted toxic effects on the rhizosphere microbial community [61,63,64,65]. The effect of AgNPs on the activity of urease, β-glucosidase, alkaline phosphatase, and the composition of the microbial community in sandy loam soils, which differ in their properties from Haplic Chernozem [64]. The effect of AgNPs on the activity of enzymes in calcareous soils, which are similar to Haplic Chernozem, was investigated [24]. The effect of similar doses of AgNPs (0.01, 0.1, 0.5, 1, 5, 10, 20, 50 mg/kg) on the activity of soil urease and phosphatase was investigated [23]. At the same time, small doses of NPs had a stimulating effect, while higher doses of 20 and 50 mg/kg inhibited the activity of enzymes.

The effect of similar doses of AgNPs as in our study 1, 10, and 100 mg/kg on the activity of phenoloxidases and arylamidase [63]. The highest ecotoxicity was shown by a dose of 100 mg/kg. It was found the inhibitory effect of AgNPs of the same size 10 nm on the activity of acid phosphatase, glucosaminidase, glucosidase, and arylsulfatase [22]. In a study, a similar dose of 1 mg/kg of AgNPs caused reductions in microbial biomass, while nitrogen-fixing bacteria were most affected [60]. Our study is consistent with the hypothesis that the higher the concentration of AgNPs in the soil, the more pronounced the toxic effect on the biological parameters of soils (enzyme activity, the number of soil bacteria, phytotoxic indicators). Earlier dose-dependent inhibitory effect of AgNPs on the germination of rice seeds and their subsequent growth and development on the microbial and enzymatic activities of the soil was noted [24,64].

Based on the obtained biological indicators of the state of soils when contaminated with AgNPs, an assessment was made of their information content and sensitivity. The sensitivity of the indicator was assessed by the degree of decrease in its values in the variants with contamination by AgNPs in comparison with the control. The informative value of the indicator was assessed by the tightness of the correlation coefficient (r) between the content of AgNPs in the soil and the biological indicator (*p* = 0.05). The degree of sensitivity, the biological parameters of Haplic Chernozem form the following sequence: total number of bacteria (72) > activity of dehydrogenases (81) > activity of catalase (90) > germination of radish (91) > length of radish roots (95) > abundance of bacteria of the genus *Azotobacter* (98).

The degree of informativeness of biological indicators of Haplic Chernozem form the following sequence: length of radish roots (−0.99) > abundance of bacteria of the genus *Azotobacter* (−0.95) ≥ germination of radish (−0.95) > activity of dehydrogenases (−0.92) > activity catalase (−0.82) ≥ total number of bacteria (−0.82). The most sensitive indicator for soil contamination with AgNPs is the total number of bacteria and the least—the abundance of bacteria of the genus *Azotobacter*. The most informativeness indicator for soil contamination with AgNPs is the length of radish roots, and the least is the total number of bacteria. Since some are sensitive, and other indicators are informative, it is advisable to use the integral indicator of the biological state (IIBS). It was recently found that the biological condition of Haplic Chernozem was negatively influenced by the AgNPs, nanoparticles of nickel, iron, cobalt, zinc [32,33,43].

It seems promising to continue studies of the toxic effect of AgNPs on the ecological state of soils in the following directions: To comprise of ecotoxicity of AgNPs of different sizes; to comprise of the toxicity of AgNPs for the soil of different chemical form of compounds of silver; to study the changes in soil properties under contamination with AgNPs in dynamics (from 10 till 360 days); to study of a wider range of biological indicators of soils after the pollution of AgNPs to comprise of the effect of AgNPs on different types of soils. These are distinguished by their buffering properties to pollution with heavy metals: Particle size distribution, reaction of the soil environment to the content of organic matter, etc.; to investigate of the combined effect of AgNPs pollution complicated with other pollutants (other heavy metals, petroleum hydrocarbons, antibiotics, pesticides, etc.).

## 5. Conclusions

The degree of reduction in biological properties depends on the AgNPs concentration in the soil. It was found that the total number of bacteria and enzymatic activity were the most sensitive indicators to contamination of AgNPs. At the minimum concentration of AgNPs (0.5 mg/kg), inhibited the enzymatic activity (activity of catalase and dehydrogenases) by 18–38% relative to the control was observed. The total number of bacteria is a more informative and sensitive indicator to AgNPs contamination than the *Azotobacter* sp. abundance. The abundance of bacteria of the genus *Azotobacter* sp. was the least sensitive indicator to contamination with AgNPs. In this regard, even at the dosage of 100 mg/kg, no decrease was evidenced. Phytotoxicological indicators (germination rate and root length of radish) demonstrated a change under the influence of low doses of 1 and 5 mg/kg of AgNPs. Even at a dose of 1 mg/kg, the length of radish roots decreased by 21%, which is an indicator of the sensitivity of this parameter. As the concentration of AgNPs increases, the degree of exposure increases. Contamination by AgNPs led to the reduction in the integrated index of biological state (IIBS) of Haplic Chernozem. It showed that AgNPs in 10 mg/kg concentration caused a decrease in the indicator by 13% (*p* < 0.05) relative to the control. In concentrations of 50 and 100 mg/kg AgNPs, IIBS were decreased by 22 and 27% (*p* < 0.05) relative to the control, respectively. Thus, the higher the concentration of AgNPs, the higher the decrease of IIBS.

## Figures and Tables

**Table 1:** Characteristics of biological indicators of soil condition.

No	Biological Indicators	Measure Unit	Methods
1	total number of bacteria	10^9^ bacteria in gram of soil dry weight	luminescent microscopy with solution of acridine orange, 40X
2	*Azotobacter* sp. abundance	% of the mud balls surrounded by *Azotobacter* mucus	the method of fouling lumps on the Ashby medium
3	catalase activity	ml O_2_ per gram of soil dry weight in 1 min.	by the rate of decomposition of hydrogen peroxide
4	dehydrogenases activity	mg of triphenylformazane (TPF) per gram of soil dry weight for hour	according to the rate of conversion of triphenyltetrazolium chloride (TPC) to TPF
5	the germination rate of radish seeds	% of germination seeds of control	germination of radish (*Raphanus sativus* L.) after 7 days of the experiment
6	the length of the radish roots	millimeters	of length of the roots in radish (*Raphanus sativus* L.) after 7 days of the experiment

**Table 2:** Change in Biological indicator of Haplic Chernozem by AgNPs pollution.

Biological Indicator	Concentration of AgNPs, mg/kg						
	Control	0.5	1	5	10	50	100
total number of bacteria, 10^9^ in gram of soil dry weight	5.1 ± 0.3	4.9 ± 0.2	4.5 ± 0.4	4.0 ± 0.3	3.2 ± 0.2	3.0 ± 0.2	2.5 ± 0.2
Azotobacter sp. abundance, % fouling lumps of soil dry weight	100.0 ± 2.0	100.0 ± 2.0	100.0 ± 2.0	100.0 ± 2.0	98.0 ± 2.0	97.0 ± 2.0	95.0 ± 2.0
catalase activity, ml O_2_ per gram of soil dry weight in 1 min	11.2 ± 1.3	9.2 ± 2.2	8.9 ± 1.7	8.6 ± 2.0	8.1 ± 1.3	7.7 ± 1.4	6.7 ± 1.0
dehydrogenases activity, mg of triphenylformazane (TPF) per gram of soil dry weight for hour	28.8 ± 1.5	17.8 ± 1.3	17.0 ± 1.2	13.4 ± 1.2	11.3 ± 1.0	9.0 ± 1.3	5.8 ± 1.0
the length of the radish roots, millimeters	68.0 ± 2.2	65.2 ± 2.6	54.0 ± 2.0	50.0 ± 2.0	40.0 ± 2.5	35.0 ± 2.0	24.0 ± 1.2
the germination rate of radish seeds, % of germination of control seeds	100.0 ± 1.4	96.0 ± 2.5	88.0 ± 3.2	80.0 ± 2.2	78.0 ± 1.8	71.0 ± 1.7	60.0 ± 3.1

## Data Availability

The data presented in this study is available upon request from the respective author. The data are not publicly available as they will be part of Natalia Tcepina’s doctoral dissertation.
